# Comparative Proteomic Profiling of Responses to Standard Systemic Treatment Regimens in Pancreatic Cancer

**DOI:** 10.3390/cells15060531

**Published:** 2026-03-17

**Authors:** Amirsalar Mansouri, Olivia Hart, Sina Aslanabadi, Conner Hartupee, Dicle Yalcin, Garima Sinha, Chiswili Yves Chabu, Aleksandra Cios, Zetao Cheng, Sudhakar Ammanamanchi, Jovanny Zabaleta, John H. Stewart, John T. West, Mitesh J. Borad, Bolni Marius Nagalo, Jiri Adamec, Omeed Moaven

**Affiliations:** 1 Department of Interdisciplinary Oncology, Louisiana State University (LSU) Health School of Medicine-New Orleans, New Orleans, LA 70112, USA; amans3@lsuhsc.edu (A.M.); saslan@lsuhsc.edu (S.A.); dyalci@lsuhsc.edu (D.Y.); samman@lsuhsc.edu (S.A.); jzabal@lsuhsc.edu (J.Z.); jwest6@lsuhsc.edu (J.T.W.); jadame@lsuhsc.edu (J.A.); 2 LSU-LCMC Health Cancer Center, New Orleans, LA 70112, USA; ohart@lsuhsc.edu; 3 Department of Internal Medicine, Tulane University, New Orleans, LA 70118, USA; chartupee@tulane.edu; 4 Division of Surgical Oncology, Department of Surgery, Louisiana State University (LSU) Health-New Orleans, New Orleans, LA 70112, USA; gsinha3397@gmail.com; 5 Division of Biological Sciences, University of Missouri, Columbia, MO 65211, USA; chabuc@missouri.edu; 6 Marlene and Stewart Greenebaum NCI Comprehensive Cancer Center, School of Medicine, University of Maryland, Baltimore, MD 21201, USA; acios@som.umaryland.edu (A.C.); zcheng@som.umaryland.edu (Z.C.); bnagalo@som.umaryland.edu (B.M.N.); 7 Department of Pharmacology and Physiology, University of Maryland, Baltimore, MD 20740, USA; 8 Department of Surgery, Morehouse School of Medicine, Atlanta, GA 30310, USA; jstewart@msm.edu; 9 Division of Hematology/Oncology, Department of Internal Medicine, Mayo Clinic, Phoenix, AZ 85259, USA; borad.mitesh@mayo.edu

**Keywords:** PDAC, MIA PaCa2, FOLFIRINOX, gemcitabine, chemotherapy, proteomics

## Abstract

**Highlights:**

**What are the main findings?**

- FOLFIRINOX and gemcitabine/nab-paclitaxel induced distinct tumor-cell-intrinsic proteomic programs in PDAC cells.
- FOLFIRINOX suppressed ribosome biogenesis and mitochondrial translation, whereas gemcitabine/paclitaxel disrupted mitotic cytokinesis and lipid signaling.

**What are the implications of the main findings?**

- First-line PDAC chemotherapies are not biologically interchangeable despite having similar clinical efficacy.
- Regimen-specific molecular pathways provide a framework for developing predictive biomarkers of chemotherapy response.

**Abstract:**

Pancreatic ductal adenocarcinoma (PDAC) is an aggressive malignancy with a 5-year survival rate of 13.3%. First-line treatment relies on two chemotherapy regimens, FOLFIRINOX (FOLFNX) or gemcitabine plus nab-paclitaxel (GEMPAC). However, direct clinical comparisons between these regimens have yielded inconsistent results across survival and toxicity endpoints, and the molecular basis of heterogeneous treatment responses remains poorly defined. To investigate regimen-specific tumor-cell-intrinsic mechanisms, we performed quantitative proteomic profiling of a primary PDAC-derived MIA PaCa-2 cell line following treatment with FOLFNX or GEMPAC. Differentially expressed proteins were analyzed using Gene Ontology, KEGG, and Ingenuity Pathway Analysis to define pathway-level alterations, and findings were contextualized using TCGA transcriptomic data. Proteomic analyses revealed that FOLFNX and GEMPAC engage in distinct cytotoxic programs. FOLFNX predominantly suppressed ribosome biogenesis and mitochondrial translation, consistent with sustained metabolic and biosynthetic stress, whereas GEMPAC preferentially disrupted mitotic cytokinesis and phosphatidylinositol phosphate biosynthesis, consistent with mitotic failure. Integration with TCGA data showed that FOLFNX-altered proteins aligned with favorable prognostic expression signatures, whereas GEMPAC-associated proteins were enriched among adverse profiles, reflecting engagement of distinct tumor-intrinsic programs. Together, these findings provide mechanistic insight into differential chemotherapy responses and establish a foundation for proteomics-based biomarkers to guide personalized chemotherapy selection in PDAC.

## 1. Introduction

Pancreatic ductal adenocarcinoma (PDAC) is among the most lethal cancer types worldwide, with a 5-year survival rate of only 13.3% [1]. PDAC is projected to become the second leading cause of cancer-related deaths in the United States by 2030 due to rising incidence rates [2]. At the time of diagnosis, the majority of PDAC patients present with advanced disease, only 15–20% of whom have resectable tumors. Even among the patients who undergo surgery, the 5-year survival rate remains low at approximately 20%, largely due to early systemic dissemination, occult micrometastatic disease, and rapid post-operative recurrence [3,4].

The current standard of care for systemic treatment of PDAC is chemotherapy (CT) using one of two regimens: FOLFIRINOX (FOLFNX) or Gemcitabine plus Nab-paclitaxel (GEMPAC) [3]. FOLFNX is a combination therapy comprising oxaliplatin, irinotecan, leucovorin, and 5-fluorouracil, while GEMPAC combines Gemcitabine with nanoparticle albumin-bound paclitaxel.

In separate clinical trials comparing each regimen to Gemcitabine monotherapy, FOLFNX achieved a median overall survival (mOS) of 11.1 months while GEMPAC produced an mOS of 8.5 months [5,6]. Several recent phase II clinical trials have directly compared FOLFNX and GEMPAC for the treatment of metastatic and locally advanced pancreatic cancer, revealing comparable efficacy but regimen-specific toxicity profiles that vary across clinical trials and patient populations, reflecting differences in study design, patient selection, and toxicity assessment. The PASS-01 trial concluded that progression-free survival (PFS) was similar between FOLFNX and GEMPAC, but GEMPAC provided superior overall survival (OS) and safety profile in metastatic pancreatic cancer [7]. The Generate trial revealed a longer OS with GEMPAC (17.1 months) than FOLFNX (14 months) in metastatic or recurrent pancreatic cancer; however, the trial was terminated early due to its low predictive value [8]. A 2021 randomized phase II trial showed a longer 1-year OS with GEMPAC but a better 2-year OS with FOLFNX in locally advanced pancreatic cancer [9]. In several clinical trials and retrospective studies comparing FOLFNX and GEMPAC for neoadjuvant therapy, the two regimens have demonstrated similar overall survival, rates of R0 resection, and safety for resectable and borderline-resectable pancreatic cancer [10,11,12,13,14,15].

In clinical practice, regimen selection is often guided by a patient’s Eastern Cooperative Oncology Group Performance Status (ECOG PS). FOLFNX is often preferred in younger patients with lower ECOG PS, whereas GEMPAC is considered for older patients who may not tolerate FOLFNX well [16]. Comparisons of toxicity between FOLFNX and GEMPAC have shown inconsistent results. Overall, treatment with FOLFNX is associated with higher rates of neutropenia, while GEMPAC is associated with increased anemia and neurotoxicity [16,17,18]. The paucity of direct comparative trials between FOLFNX and GEMPAC, along with the variability in survival and toxicity data, underscores a fundamental gap in understanding their therapeutic effects.

The observed variability in response indicates that these regimens target distinct biological vulnerabilities across patients. Biomarkers that capture these differences may inform treatment selection and optimize therapeutic benefit. Alterations in protein expression could provide a direct reflection of treatment response, revealing chemotherapy’s effects on key signaling pathways, metabolism, and immune-related processes that may contribute to differences in response. Proteomic analysis of tumor cells, therefore, has the potential to identify treatment-associated molecular programs that may inform future development of predictive biomarkers and more personalized therapeutic strategies for PDAC.

MIA PaCa-2 cells, a primary tumor-derived PDAC cell line, are widely accepted as an in vitro model due to their (i) hallmark KRAS, TP53, and CDKN2A mutations, (ii) epithelial–mesenchymal phenotype, (iii) intrinsic population heterogeneity, and (iv) multidrug sensitivity [19,20,21]. Here, we investigated treatment-induced alterations in the cellular proteomes following either FOLFNX or GEMPAC treatment, enabling direct assessment of tumor-cell-intrinsic molecular responses independent of stromal or immune influences. These characteristic PDAC features provide a robust system to dissect treatment-specific molecular adaptations to FOLFNX and GEMPAC.

In addition, cross-referencing the treatment-induced signature proteins with Gene Ontology (GO), Ingenuity Pathway Analysis (IPA), Kyoto Encyclopedia of Genes and Genomes (KEGG), and The Cancer Genome Atlas (TCGA) datasets allows for the identification of key molecular pathways affected by each treatment regimen. By characterizing therapy-associated cellular changes, this proteomic analysis aims to advance understanding of differential treatment responses in PDAC and support future efforts toward more individualized therapeutic approaches.

## 2. Materials and Methods

### 2.1. Study Design

MIA PaCa2 PDAC cells were prepared according to the procedures outlined below and allocated into one of three groups: untreated controls (CTRL), FOLFNX-treated, or GEMPAC-treated. For clarity, the in vitro FOLFNX condition represents a modified FOLFIRINOX-like combination excluding leucovorin. Following a 10 h exposure, cells were harvested for protein extraction. Isolated proteins were analyzed using Liquid Chromatography–Mass Spectrometry (LC-MS) for quantitative proteomic profiling. Differentially expressed proteins were identified through three pairwise comparisons: FOLFNX vs. CTRL, GEMPAC vs. CTRL, and FOLFNX vs. GEMPAC. Dysregulated proteins were subsequently interrogated using KEGG pathway enrichment, Gene Ontology (GO) analysis, Ingenuity Pathway Analysis (IPA), and protein–protein interaction (PPI) network construction.

Finally, treatment-specific proteins of interest were integrated with TCGA PAAD datasets to assess prognostic alignment and contextualize clinical relevance.

### 2.2. Cell Preparation and Dose Optimization

MIA PaCa-2 cells were procured from the American Type Culture Collection (ATCC). Cells were cultured in Dulbecco’s Modified Eagle Medium (DMEM) supplemented with 10% fetal bovine serum (Sigma-Aldrich F2442, Burlington, MA, USA) and grown to 70–90% confluency in 6-well culture dishes at 37 °C with 5% CO_2_.

To define treatment concentrations for downstream proteomic analysis, dose–response viability assays were performed prior to experimental treatment. For viability assays, cells were seeded in 96-well plates at 3000–5000 cells per well and allowed to adhere overnight to ensure exponential growth during treatment. Serial dilutions of gemcitabine, paclitaxel, 5-fluorouracil, irinotecan, and oxaliplatin were prepared in culture medium.

For the Gem/Pac combination (GEMPAC), the reference (1×) concentration was defined as 20 μM gemcitabine + 0.4 μM paclitaxel. For FOLFIRINOX (FOLFNX), the reference (1×) concentration was defined as 4 μM 5-fluorouracil + 2 μM irinotecan + 4 μM oxaliplatin. Three relative concentrations (0.1×, 1×, and 10× of reference) were tested for each regimen. Cells were treated for 24 h prior to viability assessment.

Cell viability was quantified in six replicate wells per condition using the MTS assay (CellTiter 96^®^ AQueous One Solution Cell Proliferation Assay, Promega, Madison, WI, USA) according to the manufacturer’s instructions. Both regimens demonstrated graded, concentration-dependent reductions in viability (Supplementary Figure S1). The 1× concentration produced intermediate (~40–60%) viability reduction and was selected for proteomic experiments to induce substantial but incomplete cytotoxic stress. The selected in vitro concentrations were based on IC50-range viability optimization and do not directly reflect pharmacokinetic plasma concentrations achieved in patients.

For proteomic experiments, cells were treated for 10 h at 37 °C with the optimized chemotherapeutic concentrations. A 10 h exposure time was selected to interrogate early chemotherapy-induced molecular remodeling prior to the onset of widespread apoptosis. Preliminary live-cell imaging experiments indicated that overt cytotoxic morphological changes emerged after approximately 12–16 h, supporting the selection of an earlier timepoint for proteomic analysis.

The FOLFNX condition consisted of 5-fluorouracil (4 µM), irinotecan (2 µM), and oxaliplatin (4 µM). Leucovorin (folinic acid), which functions clinically as a biochemical modulator of 5-fluorouracil rather than an independent cytotoxic agent, was not included in the in vitro treatment regimen. The GEMPAC condition consisted of gemcitabine (20 µM) and paclitaxel (0.4 µM).

### 2.3. Protein Extraction and Digestion

For protein extraction, MIA PaCa2 cell samples were treated with an equal volume (in µL) of ABC Buffer (100 mM Ammonium Bicarbonate, pH 8) and eight volumes of 100% Methanol. Samples underwent homogenization using a Bead Ruptor 96 (OMNI International, Kennesaw, GA, USA) with six large beads. The resulting suspensions (180 µL) were transferred to new Eppendorf tubes, centrifuged at 14,000× *g* for 5 min, and the supernatant was discarded. Pellets were washed twice with cold acetone and dried using a SpeedVac concentrator (Thermo Fisher Scientific, Waltham, MA, USA). For digestion, protein pellets were solubilized in 20 µL of Denaturation Buffer (25 mM ammonium bicarbonate, pH 8.0; 10 mM TCEP; 5% SDC [sodium deoxycholate]), incubated for 10 min at 60 °C, and alkylated with 5 µL of freshly prepared Alkylation Buffer (100 mM Iodoacetamide in water). Incubation continued for an additional 60 min at room temperature in the dark. Post-alkylation, samples were diluted with 175 µL of dilution buffer (25 mM ammonium bicarbonate, pH 8.0), and 2 µL of Trypsin solution (1 µg/µL) was added. Samples were incubated overnight at 37 °C. To halt the reaction and remove SDC, 10 µL of 10% TFA was added, and the mixture was incubated at room temperature for 30 min, then centrifuged at 15,000× *g* (SDC precipitates at low pH). Supernatants were then transferred to new tubes for direct LC-MS analysis.

### 2.4. LC-MS/MS Analysis

All analyses were performed using a Bruker nanoElute2 system coupled to a timsTOF fleX 2 mass spectrometer (Bruker, Billerica, MA, USA). Mobile phases consisted of Solvent A (0.1% formic acid in 3% acetonitrile) and Solvent B (0.1% formic acid in acetonitrile). Samples were injected at a volume of 2 µL. After injection, peptides were separated using a C-18 reversed-phase PepSep column (0.150 × 250 mm; 1.5 µm particle size; Bruker, Billerica, MA, USA) connected to the CaptiveSpray ionization source. The column chamber was maintained at a flow rate of 800 nL/min and a temperature of 50 °C. Peptide separation was achieved using the following gradient: T = 0 min: 0% B; T = 40 min: 26% B; T = 40.5 min: 95% B; T = 41.5 min: 95% B; T = 42 min: 0% B; T = 45 min: 0% B (column re-equilibration). Mass spectrometry data were collected in positive, Data Dependent Acquisition (DDA)—Parallel Accumulation-Serial Fragmentation (PASEF) mode under the following conditions: a capillary voltage of 1500 V; source temperature of 180 °C; dry gas flow at 3 L/min; and an acquisition range of 100–1700 *m*/*z*. The time settings were as follows: 1/K0 Start: 0.60 Vs/cm^2^; 1/K0 End: 1.60 Vs/cm^2^; Ramp Time: 100 ms; Accumulation Time: 100 ms; Duty Cycle: 100%; and Ramp Rate: 9.42 Hz.

### 2.5. Data Analysis

Proteomic data were processed using MaxQuant [22], leveraging the UniProt *Homo sapiens* database to identify proteins with the label-free quantification (LFQ) method. LFQ intensities were processed and prepared using an in-house R script. For each treatment group (CTRL, FOLFNX, and GEMPAC), proteins were evaluated for reproducibility across six biological replicates. A protein was classified as detected in each group if it was present in at least 4 of 6 replicates. Missing LFQ intensity values among detected proteins were imputed using a Perseus-style “missing not at random” (MNAR) approach, replacing missing values with random draws from a Gaussian distribution left-shifted by 1.8 standard deviations and with a width of 0.3 to simulate low-abundance signals near the instrument’s detection limit. Proteins detected in at most 2 of 6 replicates were classified as undetected, and their LFQ intensity values were set to zero to indicate the absence of reliable quantification. Proteins with intermediate replicate coverage (i.e., detected in 3 of 6 replicates) did not meet criteria for identified proteins (either detected or undetected status) and were therefore excluded from downstream analysis to minimize uncertainty in detection.

Statistical analyses were conducted on identified proteins using the R limma package (limma_3.58.1) [23] to identify differentially expressed proteins, with the alpha level set at a *p*-value of less than 0.05 and absolute log2-transformed fold change (log_2_FC) > 0.585 (corresponding to FC of 1.5). Proteins identified in a pairwise comparison but detected in only one of the two groups were additionally considered differentially expressed, as the absence of detection in one condition indicates a biologically meaningful reduction below the detection limit. A protein–protein interaction (PPI) network was constructed (medium confidence score, >0.4) using the Search Tool for the Retrieval of Interacting Genes/Proteins (STRING) 11.0 [24] and then visualized using Cytoscape software 3.7.1 [25]. Network module analysis was performed using the Molecular Complex Deletion (MCODE) [26] plugin for Cytoscape. The parameters were set as degree cut-off = 2, node score cutoff = 0.2, k-core = 2, and maximum depth = 100. For downstream analysis, Ingenuity Pathway Analysis (IPA, QIAGEN Inc., Redwood City, CA, USA; accessed November 2025), KEGG pathway analysis, and Gene Ontology (GO) analysis using the DAVID Bioinformatics Resources [27,28] were used to identify enriched pathways and interpret underlying biological processes.

## 3. Results

**Figure 1:**
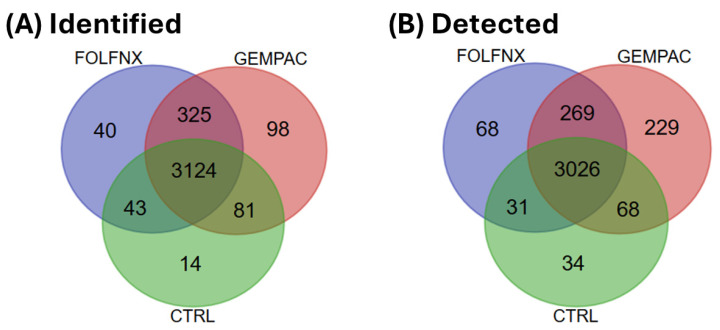
Venn diagram of proteins across FOLFIRINOX (FOLFNX), Gemcitabine + Nab-paclitaxel (GEMPAC), and untreated control (CTRL) conditions: (**A**) Identified (detected and undetected) proteins. (**B**) Detected proteins.

Across all three groups, a total of 3124 proteins were commonly identified in FOLFNX-, GEMPAC-, and CTRL-treated cells (Figure 1A). When restricted to detected proteins, 3026 proteins were shared across all conditions (Figure 1B). The list of proteins and their corresponding status for each group is included in Supplementary Table S1. To further contextualize the translational relevance of treatment-associated proteins, we annotated the proteins listed in Supplementary Table S1 for subcellular localization using UniProt annotations, including extracellular localization and signal peptide features. As expected for a cellular proteomics workflow, most proteins were intracellular and thus more suitable for tissue-based assessment, while membrane-associated or secreted proteins represent potential plasma-accessible candidates for future validation.

**Figure 2:**
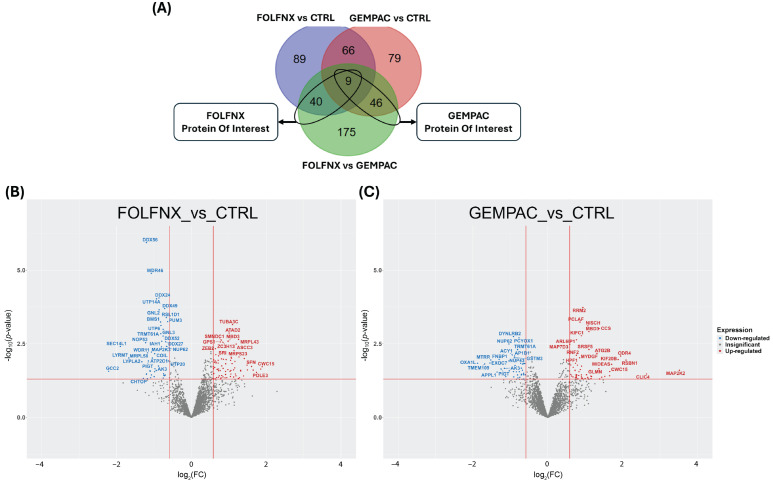
Venn diagram and volcano plots depicting commonly detected proteins significantly altered by FOLFNX and GEMPAC treatments compared to the control sample. (**A**) Venn diagram illustrates the overlap of significantly dysregulated proteins among FOLFNX, GEMPAC, and control conditions. The diagram summarizes the number of proteins significantly altered (*p* < 0.05) in each comparison: FOLFNX vs. CTRL (blue), GEMPAC vs. CTRL (red), and FOLFNX vs. GEMPAC (green). The two highlighted elliptical regions denote regimen-specific proteins of interest. (**B**) Volcano plots of relative protein levels of FOLFNX compared to the control cell (127 upregulated and 77 downregulated proteins). (**C**) Volcano plots of relative protein levels of GEMPAC compared to the control cell (141 upregulated and 59 downregulated proteins).

In pairwise analysis, the relative expression levels of the identified proteins were compared between each treatment group and the control group. Comparing FOLFNX to CTRL and GEMPAC to CTRL revealed 204 and 200 significantly dysregulated proteins, respectively (Figure 2A). Among the significantly dysregulated proteins in FOLFNX, 127 were upregulated and 77 were downregulated, while in GEMPAC 141 proteins were upregulated and 59 were downregulated. In addition to assessing treatment effects relative to CTRL, direct comparison of the two chemotherapy regimens (FOLFNX vs. GEMPAC) identified 270 significantly dysregulated proteins. Importantly, proteins dysregulated in at least two pairwise comparisons highlight treatment-specific signatures. FOLFNX-POI were defined as proteins altered in both FOLFNX vs. CTRL and FOLFNX vs. GEMPAC, while GEMPAC-POI were defined as proteins altered in both GEMPAC vs. CTRL and GEMPAC vs. FOLFNX (Figure 2A). These proteins are not only regulated relative to CTRL but also differ significantly from the alternative treatment condition, making them strong candidates for regimen-specific effects. To further characterize treatment-specific expression patterns, volcano plots were generated for commonly detected proteins in both FOLFNX vs. CTRL and GEMPAC vs. CTRL (Figure 2B,C). Together, these sets of proteins reveal both shared and unique proteomic signatures associated with FOLFNX and GEMPAC chemotherapy treatments relative to untreated control cells. Supplementary Tables S2–S4 provide log2 fold changes and statistical results for the FOLFNX vs. CTRL, GEMPAC vs. CTRL, and FOLFNX vs. GEMPAC comparisons, respectively.

### 3.1. Pathway and Functional Analysis

**Figure 3:**
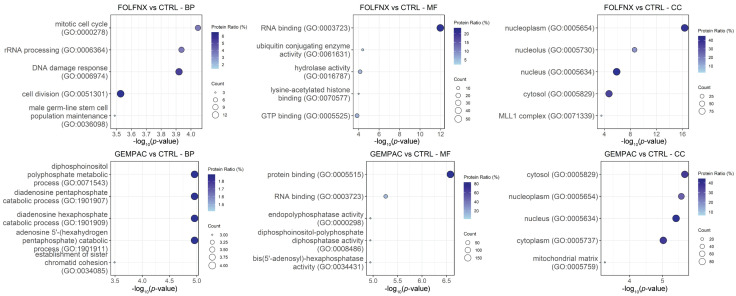
Top five Gene Ontology (GO) terms enriched among significantly dysregulated proteins in FOLFNX- and GEMPAC-treated MIA PaCa-2 cells. Panels display the top five enriched GO terms (*p* < 0.05) for each ontology category: Biological Process (BP), Molecular Function (MF), and Cellular Component (CC), arranged from left to right. The (**top row**) shows enrichment results for FOLFNX vs. CTRL, and the (**bottom row**) shows GEMPAC vs. CTRL. Dot size corresponds to the number of proteins associated with each GO term, and color intensity reflects the gene ratio, defined as the percentage of significantly dysregulated proteins mapping to that term.

To characterize the functional programs affected by chemotherapy treatment, Gene Ontology (GO) enrichment analysis was performed on proteins significantly dysregulated in FOLFNX- and GEMPAC-treated MIA PaCa-2 cells relative to untreated controls. Distinct patterns of enrichment were observed across Biological Process (BP), Molecular Function (MF), and Cellular Component (CC) categories, indicating that the two regimens induce different tumor-cell–intrinsic functional responses. In FOLFNX-treated cells, enriched BP terms were dominated by cell-cycle regulation, DNA damage response, and RNA processing pathways, consistent with broad suppression of proliferative and biosynthetic programs. Correspondingly, MF terms highlighted enrichment of RNA binding and enzymatic activities involved in nucleic acid metabolism, while CC terms were strongly associated with nuclear and nucleolar compartments, reflecting disruption of transcriptional and ribosomal machinery. In contrast, GEMPAC-treated cells exhibited enrichment of BP terms related to phosphoinositide metabolism and chromatin organization, MF terms associated with phosphatase and RNA binding activities, and CC terms linked to cytosolic and mitochondrial compartments, suggesting altered signaling and metabolic adaptation rather than generalized transcriptional suppression. These regimen-specific enrichment patterns are summarized in Figure 3, and the complete GO enrichment results for FOLFNX and GEMPAC, compared with CTRL, are provided in Tables S5–S7 and Tables S8–S10, respectively.

To further interpret the biological and functional relevance of proteins significantly altered by FOLFNX and GEMPAC treatment, pathway enrichment analyses were performed using KEGG and Ingenuity Pathway Analysis (IPA). KEGG pathway analysis revealed both shared and regimen-specific pathway perturbations. Pathways common to both treatments included nucleotide excision repair, DNA replication, and cell-cycle regulation, reflecting shared induction of genotoxic and replication stress. However, distinct pathway signatures emerged for each regimen.

**Figure 4:**
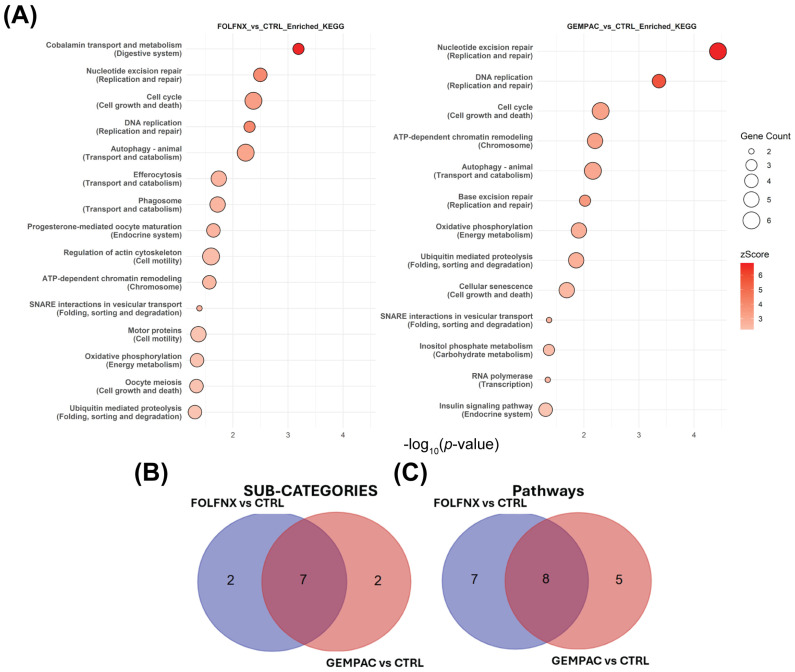
KEGG pathway enrichment and overlap analysis in FOLFNX- and GEMPAC-treated MIA PaCa-2 cells. (**A**) Dot plots of significantly enriched KEGG pathways (*p* < 0.05) for FOLFNX vs. CTRL (**left**) and GEMPAC vs. CTRL (**right**). Dot size indicates the number of mapped proteins, and color represents KEGG z-scores. Human Disease pathways were excluded; complete lists of pathways are provided in Supplementary Tables S11 and S12. Venn diagrams show the overlap of enriched KEGG (**B**) sub-categories and (**C**) individual KEGG pathways between FOLFNX and GEMPAC.

In FOLFNX-treated cells, KEGG enrichment highlighted pathways related to cobalamin transport and metabolism, autophagy, phagosome formation, and chromatin remodeling, consistent with suppression of biosynthetic capacity and cellular stress responses. In contrast, GEMPAC-treated cells were characterized by enrichment of pathways involved in ATP-dependent chromatin remodeling, oxidative phosphorylation, inositol phosphate metabolism, and vesicular transport, suggesting altered mitotic control and metabolic reprogramming. These enriched pathways are shown in Figure 4A, while overlap analysis at both the KEGG subcategory and individual pathway levels (Figure 4B,C) demonstrates limited overlap between FOLFNX and GEMPAC, underscoring their divergent molecular effects.

**Figure 5:**
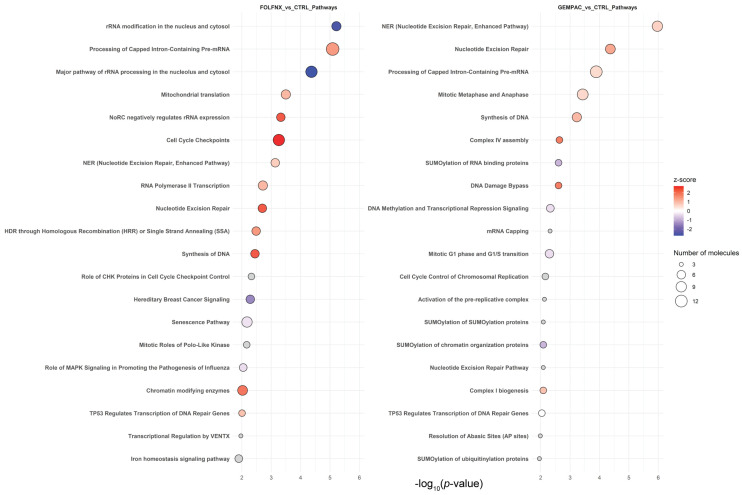
Top 20 IPA canonical pathways enriched from proteins significantly dysregulated by FOLFNX (**left**) or GEMPAC (**right**) treatment compared with control samples in MIA PaCa-2 pancreatic cancer cells. Pathways are ranked by statistical significance (−log_10_ *p*-value). Dot size denotes the number of differentially expressed proteins mapping to each pathway, and dot color represents IPA z-scores, indicating predicted pathway activation (red) or inhibition (blue).

IPA canonical pathway analysis further supported these findings, identifying coherent but distinct pathway activation patterns for each treatment (Figure 5). FOLFNX-enriched IPA pathways predominantly involved RNA processing, DNA damage signaling, and mitochondrial dysfunction, whereas GEMPAC-enriched pathways emphasized mitotic regulation, lipid signaling, and metabolic adaptation. Complete KEGG and IPA pathway enrichment results are provided in Supplementary Tables S11 and S12 (KEGG) and Supplementary Tables S13 and S14 (IPA), respectively. Together, KEGG and IPA pathway analyses converge on the conclusion that FOLFNX and GEMPAC engage distinct tumor-cell-intrinsic biological programs despite overlapping induction of core stress responses.

### 3.2. Protein–Protein Interaction Networks

To identify coordinated functional modules uniquely remodeled by each chemotherapy regimen, we constructed protein–protein interaction (PPI) networks using the treatment-specific POI. As defined earlier, FOLFNX-POI represent proteins significantly dysregulated in both FOLFNX vs. CTRL and FOLFNX vs. GEMPAC. In contrast, GEMPAC-POI represents proteins significantly dysregulated in both GEMPAC vs. CTRL and GEMPAC vs. FOLFNX. Focusing on these POI sets enabled the PPI analysis to capture regimen-specific molecular signatures, rather than the global treatment effects previously captured in pathway analysis.

**Figure 6:**
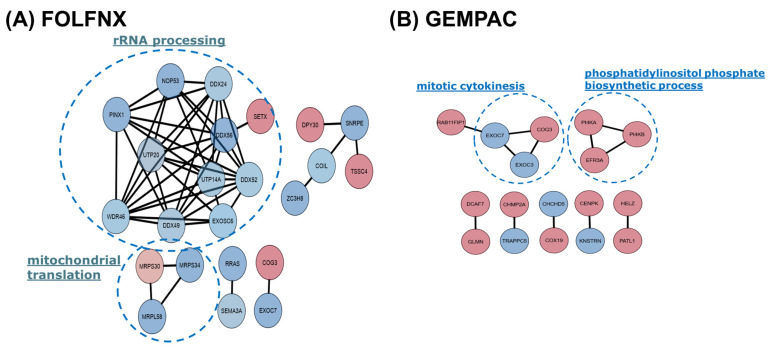
Protein–protein interaction (PPI) network and module analysis of FOLFNX- and GEMPAC-POI. PPI networks were constructed using STRING v11.0 (confidence > 0.4), visualized in Cytoscape v3.7.1, and clustered using MCODE (degree cut-off = 2, node score cutoff = 0.2, k-core = 2, maximum depth = 100). (**A**) PPI network of FOLFNX-POI, and (**B**) PPI network of GEMPAC-POI. Node color denotes differential protein expression relative to controls: red indicates upregulated proteins, while blue indicates downregulated proteins. Edge lines represent interactions supported by STRING evidence. Dashed circles highlight the MCODE-defined clusters representing the most functionally enriched protein modules for each chemotherapy condition.

Using the STRING database (medium confidence > 0.4) and Cytoscape visualization followed by MCODE module detection, we identified four connected clusters enriched for coherent biological processes (Figure 6). Relevant biological processes of these clusters found using DAVID revealed that FOLFNX-induced clusters were strongly associated with rRNA processing and mitochondrial translation (Figure 6A). In contrast, GEMPAC-specific modules highlighted distinct processes, including mitotic cytokinesis and phosphatidylinositol phosphate biosynthesis (Figure 6B).

### 3.3. TCGA Integration

To assess the clinical relevance of treatment-specific proteomic changes, we cross-referenced the FOLFNX- and GEMPAC-derived POI with prognostic genes identified in the TCGA pancreatic adenocarcinoma (PAAD) cohort metadata study, which includes transcriptomic data from 176 patients [29,30,31]. Proteins were classified into two outcome-associated groups based on their directionality and prognostic annotation: a Treatment-Induced Beneficial Prognostic Profile, comprising downregulated unfavorable and upregulated favorable genes, and a Treatment-Induced Adverse Prognostic Profile, comprising downregulated favorable and upregulated unfavorable genes. TCGA survival associations were derived using publicly available prognostic annotations from the Human Protein Atlas pancreatic adenocarcinoma resource. Prognostic classification was based on median dichotomization of gene expression levels (high vs. low expression) followed by Kaplan–Meier survival analysis and log-rank testing. Hazard ratios were calculated using Cox proportional hazards regression models as reported in the referenced TCGA analyses. As shown in Table 1, several FOLFNX-POIs—including DNAJC21, UTP14A, FNDC3B, and RRAS—mapped to the Beneficial Prognostic Profile, indicating a treatment-driven shift toward expression patterns associated with improved survival in TCGA. In contrast, MAGED2 and the validated prognostic markers S100A13 and PATL1 fell into the Adverse Prognostic Profile. For GEMPAC, only KNSTRN and PI4KB aligned with the Beneficial Prognostic Profile, whereas a larger set of proteins—including PEX13, MBNL2, ASF1B, PATL1, CENPK, WDR44, and COX19—mapped to the Adverse Prognostic Profile. In Table 1, genes shown in bold also appeared within the regimen-specific PPI modules, linking prognostic associations to coordinated functional networks, while underlined genes correspond to validated prognostic markers from TCGA. A complete list of all dysregulated proteins across the three pairwise comparisons, along with their corresponding TCGA prognostic classifications, is provided in Supplementary Table S15. These findings indicate that FOLFNX and GEMPAC differ in the prognostic directionality of their tumor-cell-intrinsic molecular effects, as assessed by directional alignment of regimen-specific POIs with TCGA-PAAD prognostic annotations. Specifically, FOLFNX-POIs more frequently corresponded to expression patterns linked to favorable patient outcomes, whereas GEMPAC-POI were enriched for proteins associated with adverse prognosis. Importantly, TCGA integration was not intended to classify MIA PaCa-2 cells into prognostic subtypes at baseline or to infer transcriptomic shifts following treatment. Rather, TCGA data were used as a contextual framework to evaluate whether treatment-altered POIs align with molecular programs associated with patient outcomes in human PDAC. As transcriptomic profiling of untreated or treated cells was not performed, multi-omics concordance and subtype reassignment remain important areas for future investigation.

## 4. Discussion

This study presents proteomic alterations in MIA PaCa2 cells after treatment with the two current first-line chemotherapeutic regimens for PDAC, FOLFNX and GEMPAC, to gain insights into the biological mechanisms they distinctly contribute to treatment response variability. While clinical outcomes suggest similar overall survival between the two regimens, direct comparison has yielded inconsistent results, and the molecular basis for differential efficacy and toxicity in individual patients remains poorly characterized [4,7,13,32]. This study was designed as a controlled in vitro proteomic analysis to delineate regimen-specific molecular responses within a defined PDAC genetic background rather than to establish universal PDAC-wide signatures.

Therapeutic options for PDAC beyond chemotherapy are limited, as the majority of tumors are unresectable at diagnosis, and a highly desmoplastic, immunosuppressive tumor microenvironment limits response to immunotherapeutic approaches [33,34]. In the context of chemotherapy, the identification of biomarkers to inform personalized treatment decisions has proven valuable in improving efficacy while minimizing toxicity across multiple cancer types [35]. In PDAC, several studies have demonstrated differential treatment responses associated with specific genetic mutations. Notably, patients with BRCA1/2 or PALB2 mutations resulting in homologous recombination deficiency achieve improved overall survival with platinum-based chemotherapies such as FOLFNX [36,37,38,39,40]. However, these mutations occur in only 6% of PDAC cases, leaving patient performance status as the primary determinant guiding selection between FOLFNX and GEMPAC for most individuals [13,41,42].

PDAC is considered highly heterogeneous across genetic, transcriptional, phenotypic, and microenvironmental domains, resulting in variability in proliferative state, metabolic programs, and therapeutic sensitivity that underlies inconsistent treatment responses. Genetically, most PDAC tumors harbor oncogenic KRAS mutations, while somatic alterations of tumor suppressor genes TP53, CDKN2A, and SMAD4 are also common drivers of PDAC development [43,44]. In addition to these common mutations, many other genetic alterations have been identified in PDAC, such as those involved in DNA damage repair, cell cycle maintenance, and MAPK and PI3K/AKT signaling pathways [45,46]. Genetic differences in PDAC tumors may contribute to differential treatment responses, and this immense genetic heterogeneity has presented a challenge in the development of personalized therapies targeting specific mutations [46]. Transcriptomic classifications of PDAC have also been developed, categorizing tumors as either classical or basal-like (squamous) subtypes based on their gene expression profiles [47]. However, abundant heterogeneity exists within these subtypes due to differences in epigenetic regulation and the tumor microenvironment [48,49]. Together, these sources of tumor diversity likely contribute to differences in treatment response, underscoring the critical need for novel biomarkers capable of predicting response to specific chemotherapeutic approaches.

MIA PaCa2 cells harbor PDAC’s three most common hallmark genetic alterations: KRAS, TP53, and CDKN2A, mirroring the genomic heterogeneity of human tumors [19]. Phenotypically, MIA PaCa-2 contains morphologically distinct subpopulations that differ in proliferative capacity, epithelial–mesenchymal features, and sensitivity to cytotoxic stress, properties that are directly relevant to chemotherapy response [20]. Transcriptomic analyses further demonstrate heterogeneous activation of cell-cycle, metabolic, and stress-response programs within the population, underscoring tumor-intrinsic molecular variability that can influence regimen-specific effects [19]. Additionally, MIA PaCa-2 cells exhibit poor differentiation with concurrent expression of epithelial and mesenchymal markers, reflecting the aggressive growth and invasive behavior characteristics of PDAC [50,51]. Because PDAC stage is a clinical rather than tumor-cell-intrinsic property, MIA PaCa-2 models aggressive PDAC biology rather than a specific disease stage; TCGA integration was therefore used for molecular contextualization rather than stage-specific inference. Importantly, the KRAS, TP53, and CDKN2A alterations present in MIA PaCa-2 cells are all somatic tumor-derived events rather than germline mutations. These canonical PDAC driver alterations are stably maintained and widely conserved across PDAC tumors, providing a representative tumor-intrinsic genetic background for mechanistic studies. While additional genomic or transcriptional variability may arise during long-term culture, the core driver genotype of MIA PaCa-2 has been extensively characterized and remains stable, supporting its use as a reproducible model of aggressive PDAC biology.

Cross-referencing chemotherapy-induced proteomic alterations with the KEGG database highlighted molecular pathways impacted by FOLFNX and GEMPAC that are consistent with established cytotoxic mechanisms in PDAC and other solid tumors. Both regimens altered core processes essential for tumor cell survival, including DNA replication, nucleotide excision repair, and cell-cycle regulation, reflecting shared effects of genotoxic and replication stress. Regimen-specific pathway alterations were also observed, including FOLFNX-associated changes in cobalamin transport and metabolism and GEMPAC-associated perturbation of ATP-dependent chromatin remodeling (Figure 4). In cancer cells, cobalamin-related pathways are tightly linked to one-carbon metabolism, nucleotide synthesis, and mitochondrial function; thus, their disruption under FOLFNX likely reflects broader suppression of biosynthetic and metabolic capacity rather than vitamin-specific effects. Together, KEGG pathway analysis—supported by concordant IPA canonical pathway enrichment—delineated distinct but biologically coherent mechanisms by which FOLFNX and GEMPAC exert differential cytotoxic stress.

To further define chemotherapy-specific mechanisms, direct comparison of proteomic alterations was conducted across three pairwise groups: FOLFNX vs. untreated controls, GEMPAC vs. untreated controls, and FOLFNX vs. GEMPAC (Figure 2). This approach provides understanding of the proteomic alterations caused by each chemotherapeutic, revealing regimen-specific molecular mechanisms of action, while also providing insight into the similarities and differences between their mechanisms. This comparison uncovers two specific subsets of “proteins of interest” (POIs): FOLFNX-POIs were defined as those dysregulated in both FOLFNX versus control and FOLFNX versus GEMPAC comparisons. In contrast, GEMPAC-POIs were defined as those dysregulated in both GEMPAC versus control and FOLFNX versus GEMPAC comparisons. These subsets, therefore, identify proteins that are responsive to chemotherapy while also contributing to molecular differences between FOLFNX and GEMPAC responses. POIs were then cross-referenced with Gene Ontology (GO) and The Cancer Genome Atlas (TCGA) datasets to identify differential pathway alterations between regimens and to explore potential prognostic implications.

GO data was utilized to develop protein–protein interaction (PPI) networks for the two groups of POIs, highlighting molecular pathways that were differentially altered by each chemotherapeutic regimen (Figure 6). Consistent with pathway-level enrichment, the FOLFNX-POI PPI modules revealed a tightly interconnected network centered on rRNA processing, RNA metabolism, and mitochondrial translation, with nearly all proteins in these clusters downregulated. This coordinated suppression suggests impaired ribosome biogenesis and mitochondrial dysfunction as central features of the cellular response to FOLFNX, aligning with its broad genotoxic and biosynthesis-inhibiting effects. FOLFNX has previously been found to inhibit ribosome biogenesis in pancreatic cancer, and two of its major components, 5-FU and oxaliplatin, have been shown to exert cytotoxic effects by dysregulating ribosomal RNA processing [52,53,54,55]. Additionally, platinum-based drugs such as oxaliplatin have been shown to disrupt mitochondrial function; however, this mechanism has not been thoroughly studied in pancreatic cancer [56]. These discoveries therefore support our findings on the cytotoxic mechanisms of FOLFNX, and our proteomic analysis provides a more detailed understanding of the direct pathway components impacted by this chemotherapy.

In contrast to FOLFNX, the GEMPAC-POI PPI modules highlighted dysregulation of mitotic cytokinesis and a pronounced upregulation of phosphatidylinositol phosphate biosynthetic enzymes, indicating chemotherapy-driven alterations in the cell-division machinery and lipid-mediated signaling pathways. The upregulation of cytokinesis-related proteins is consistent with paclitaxel-induced mitotic stress, while increased lipid signaling reflects metabolic adaptation during aberrant mitosis. Nab-paclitaxel, a component of GEMPAC, is known to stabilize microtubules and therefore block cell cycle progression and mitotic cytokinesis, inducing apoptosis [57]. Studies have shown that Gemcitabine activates the PI3K/Akt pathway, and it is theorized that this activation may contribute to resistance to Gemcitabine [58]. PI4KA and PI4KB, two proteins that we found to be upregulated by GEMPAC, are essential substrates for the PI3K/Akt pathway [59]. The upregulation of these molecules may therefore contribute to the mostly prognostically adverse profile of GEMPAC-POIs.

Based on the convergence of GO enrichment, KEGG/IPA pathway analysis, and regimen-specific PPI network architecture, FOLFNX and GEMPAC engage distinct tumor-cell-intrinsic cytotoxic programs in MIA PaCa2 cells that suggest context-dependent utility rather than interchangeable mechanisms. At 10 h, GEMPAC was associated with dysregulation of proteins governing mitotic cytokinesis and phosphatidylinositol/lipid signaling pathways, processes characteristic of highly proliferative tumor states, raising the hypothesis that GEMPAC-associated mitotic cytokinesis signatures may be enriched in proliferative tumor-cell states. In contrast, FOLFNX was associated with coordinated downregulation of proteins involved in ribosome biogenesis and mitochondrial translation, consistent with sustained metabolic and biosynthetic stress. These non-overlapping mechanisms provide a molecular explanation for why the two regimens may not be biologically interchangeable despite comparable efficacy in some clinical settings and help rationalize heterogeneous treatment responses observed in PDAC. By resolving regimen-specific proteomic programs within a uniform cellular background, this study provides mechanistic insight into how distinct cytotoxic strategies engage aggressive PDAC biology and highlights molecular pathways that could be prioritized for future biomarker validation. While the convergence of multiple enrichment and network analyses supports coordinated pathway remodeling, these findings are based on quantitative proteomic inference and should be interpreted as hypothesis-generating rather than definitive functional confirmation.

The distinct molecular architectures revealed by the PPI networks, GO terms, and KEGG/IPA pathway enrichment analyses were further contextualized through integration of treatment-specific POIs with TCGA prognostic signatures. FOLFNX-POIs that mapped to the Treatment-Induced Beneficial Prognostic Profile—including DNAJC21, UTP14A, FNDC3B, and RRAS—were essential within PPI modules governing rRNA processing and mitochondrial translation, both of which were broadly downregulated (Figure 6A, Table 1). This convergence across PPI topology, pathway enrichment, and prognostic annotation suggests that suppression of ribosome biogenesis and mitochondrial protein synthesis may contribute not only to FOLFNX’s cytotoxicity but also to its alignment with survival-associated gene-expression patterns observed in TCGA PAAD tumors. In contrast, GEMPAC-POIs were predominantly categorized within the Treatment-Induced Adverse Prognostic Profile, including several validated TCGA markers (PATL1, COX19, MBNL2) (Figure 6B, Table 1). These proteins populated GEMPAC-POI PPI modules enriched for mitotic cytokinesis and phosphatidylinositol phosphate biosynthesis, processes that were generally upregulated by treatment. This pattern reflects a shift toward molecular signatures associated with poor patient survival, consistent with KEGG and IPA findings of enhanced mitotic signaling, SUMOylation, and lipid-regulatory pathways under GEMPAC exposure. Together, these integrated analyses demonstrate that the biological processes perturbed by each chemotherapy regimen not only differ mechanistically but also carry distinct prognostic implications.

Although the present study was conducted in a monoculture system, we hereby discuss the tumor microenvironment explicitly to delineate the boundaries of interpretation for tumor-cell-intrinsic proteomic responses and to contextualize how these findings relate to therapeutic mechanisms observed in vivo. This study intentionally focuses on tumor-cell-intrinsic responses and therefore does not capture contributions from the pancreatic tumor microenvironment, including cancer-associated fibroblasts, immune cells, endothelial cells, or extracellular matrix components that are known to influence disease progression and therapeutic response [60,61]. The absence of these elements likely impacts drug efficacy and the resulting proteomic signatures, particularly for regimens such as gemcitabine/nab-paclitaxel, whose clinical activity is influenced by stromal architecture, drug delivery barriers, and immune modulation [6]. Importantly, no single experimental system fully recapitulates human pancreatic cancer. While in vivo and organoid-based models enable interrogation of stromal and immune interactions, these approaches often rely on murine systems or simplified co-cultures that differ substantially from human PDAC in immune composition, stromal organization, and therapeutic response [62,63,64]. Conversely, tumor-cell monoculture systems sacrifice microenvironmental complexity but provide high-resolution insight into tumor-intrinsic molecular mechanisms [65,66]. Within this context, our approach complements existing PDAC models by isolating regimen-specific cytotoxic programs that can later be integrated with more complex, microenvironment-aware systems.

This study is subject to several important limitations. First, all experiments were performed in a single PDAC cell line (MIA PaCa-2), which cannot fully capture the genetic and phenotypic heterogeneity characteristic of human pancreatic tumors. Second, the in vitro system lacks the tumor microenvironment (TME), including stromal, immune, and vascular components, that profoundly modulate chemotherapy sensitivity in PDAC and contribute to treatment resistance [67,68]. These constraints limit the direct translational generalizability of our findings across the molecular and phenotypic spectrum of PDAC. Validation in additional subtype-representative cell lines, patient-derived organoids, and in vivo systems will be necessary to determine the breadth of applicability of the regimen-specific proteomic signatures identified here. At the same time, the absence of microenvironmental complexity enabled isolation of tumor-cell-intrinsic drug effects. Notably, even in the absence of stromal components that are known to influence the clinical activity of gemcitabine/nab-paclitaxel, FOLFNX and GEMPAC triggered fundamentally distinct intracellular stress and death programs. This separation of intrinsic cytotoxic mechanisms from microenvironment-mediated delivery effects provides a mechanistic framework that can be leveraged in future studies to integrate drug mechanisms with TME-aware strategies aimed at improving drug delivery, overcoming resistance, and ultimately enhancing patient outcomes. Accordingly, future work extending these analyses to additional PDAC models—including cell lines representing classical and basal-like molecular subtypes, as well as patient-derived organoids and xenograft systems—will be essential to validate the regimen-specific molecular signatures identified here and to determine their utility as predictive biomarkers in more clinically relevant contexts [69,70,71,72]. We did not directly assess transcript-level concordance for selected proteins of interest. Future studies integrating matched RNA sequencing and quantitative proteomics will be necessary to determine the degree of transcriptional versus translational regulation underlying the regimen-specific proteomic signatures identified here.

The present study focuses on early (10 h) tumor-cell-intrinsic responses and therefore does not capture later adaptive or resistance-associated proteomic remodeling that may emerge at extended treatment durations (24–96 h). Longitudinal time-course analyses will be necessary to determine whether the regimen-specific pathway alterations identified here persist, intensify, or reverse during prolonged chemotherapy exposure. Additionally, the in vitro FOLFNX regimen excluded leucovorin, which clinically functions as a modulator of 5-FU; therefore, potential pharmacokinetic or tumor-microenvironment-dependent modulation effects are not captured in this model.

Finally, this study is limited by the absence of orthogonal functional validation of the identified proteomic alterations. While mass spectrometry and pathway enrichment analyses provide robust quantitative insights, the findings remain hypothesis-generating. Future studies should incorporate targeted validation assays, including evaluation of ribosome biogenesis, mitochondrial translation and respiratory function, cytokinesis/mitotic progression, and PI4K/phosphoinositide signaling, to confirm the functional relevance of these chemotherapy-associated pathway changes in PDAC. In addition, we did not directly quantify cell-state distributions (e.g., EdU incorporation or flow cytometric cell-cycle profiling). Therefore, interpretations linking regimen-specific proteomic programs to proliferative state should be considered hypothesis-generating. Future studies integrating time-resolved proteomics with EdU incorporation and mitotic markers (e.g., phospho-histone H3) will be important to determine whether GEMPAC-associated signatures reflect selective impact on proliferative subpopulations.

In conclusion, the integration of chemotherapy-induced POI dysregulation with KEGG and GO pathway analyses provides a comprehensive framework for understanding the distinct molecular mechanisms through which FOLFNX and GEMPAC exert cytotoxic effects on PDAC. Incorporation of TCGA prognostic signatures translates these mechanistic differences into a clinically relevant context. Our findings highlight that FOLFNX and GEMPAC induce distinct pathway alterations, which may contribute to heterogeneous treatment response and toxicity profiles. The identification of regimen-specific altered pathways and proteins of interest provides a mechanistic framework for future studies aimed at developing predictive biomarkers. These observations are hypothesis-generating and require validation in additional PDAC models, including patient-derived systems, before clinical application.

## Figures and Tables

**Table 1:** Overlap of treatment-specific proteins of interest (POIs) with TCGA PAAD prognostic gene sets.

Treatment Regimen	Treatment-Induced Beneficial Prognostic Profile		Treatment-Induced Adverse Prognostic Profile	
	Downregulated Unfavorable	Upregulated Favorable	Downregulated Favorable	Upregulated Unfavorable
**FOLFNX**	DNAJC21 UTP14A * FNDC3B * RRAS *	---	MAGED2	S100A13 PATL1 *
**GEMPAC**	KNSTRN *	PI4KB *	---	PEX13 MBNL2 ASF1B PATL1 * CENPK WDR44 COX19 *

* **Bold** genes denote membership in regimen-specific PPI modules; *underlined* genes indicate validated TCGA prognostic markers.

## Data Availability

The proteomics datasets generated and/or analyzed during the current study are available in a publicly accessible repository at MassIVE dataset, under accession number [MSV000100320] (https://doi.org/10.25345/C55H7C70K, accessed on 8 March 2026). TCGA-PAAD prognostic gene lists were obtained via the Human Protein Atlas pancreatic adenocarcinoma resource (https://www.proteinatlas.org/humanproteome/cancer/pancreatic+adenocarcinoma, accessed on 8 March 2026).

## Supplementary Materials

The following supporting information can be downloaded at: https://www.mdpi.com/article/10.3390/cells15060531/s1, Figure S1: Dose–response analysis for chemotherapy concentration selection in MIA PaCa-2 cells; Table S1: Protein status; Table S2: FOLFNX vs. CTRL statistical analysis; Table S3: GEMPAC vs. CTRL statistical analysis; Table S4: FOLFNX vs. GEMPAC statistical analysis; Table S5: FOLFNX vs. CTRL GO terms—Biological Process (BP); Table S6: FOLFNX vs. CTRL GO terms—Cellular Component (CC); Table S7: FOLFNX vs. CTRL GO terms—Molecular Function (MF); Table S8: GEMPAC vs. CTRL GO terms—Biological Process (BP); Table S9: GEMPAC vs. CTRL GO terms—Molecular Function (MF); Table S10: GEMPAC vs. CTRL GO terms—Cellular Component (CC); Table S11: FOLFNX vs. CTRL KEGG pathway enrichment analysis; Table S12: GEMPAC vs. CTRL KEGG pathway enrichment analysis; Table S13: FOLFNX vs. CTRL IPA pathway analysis; Table S14: GEMPAC vs. CTRL IPA pathway analysis; Table S15: TCGA dysregulated proteins.

## Abbreviations

The following abbreviations are used in this manuscript:

PDAC	Pancreatic ductal adenocarcinoma
CT	Chemotherapy
FOLFNX	FOLFIRINOX
GEMPAC	Gemcitabine plus Nab-paclitaxel
CTRL	Untreated control
mOS	Median overall survival
PFS	Progression-free survival
OS	Overall survival
ECOG PS	Eastern Cooperative Oncology Group Performance Status
GO	Gene Ontology
IPA	Ingenuity Pathway Analysis
KEGG	Kyoto Encyclopedia of Genes and Genomes
TCGA	The Cancer Genome Atlas
LC-MS	Liquid Chromatography-Mass Spectrometry
POI	Proteins of interest
ATCC	American Tissue Culture Collection
DMEM	Dulbecco’s Modified Eagle Medium
DDA	Data Dependent Acquisition
PASEF	Parallel Accumulation-Serial Fragmentation
LFQ	Label-free quantification
MNAR	Missing not at random
PPI	Protein–protein interaction
STRING	Search Tool for the Retrieval of Interacting Genes/Proteins
MCODE	Molecular Complex Deletion
CC	Cellular Component
BP	Biological Process
MF	Molecular Function
PAAD	Pancreatic adenocarcinoma
TME	Tumor microenvironment
ECM	Extracellular matrix
