# Supplementary material for: Comparative Proteomic Profiling of Responses to Standard Systemic Treatment Regimens in Pancreatic Cancer
Source: Cells. 2026 Mar 17;15(6):531. doi: 10.3390/cells15060531 (PMC13025568; doi:10.3390/cells15060531)
Supplement: Supplementary file 1 [file cells-15-00531-s001.zip › cells-4123907-supplementary/Figure S1.pdf]

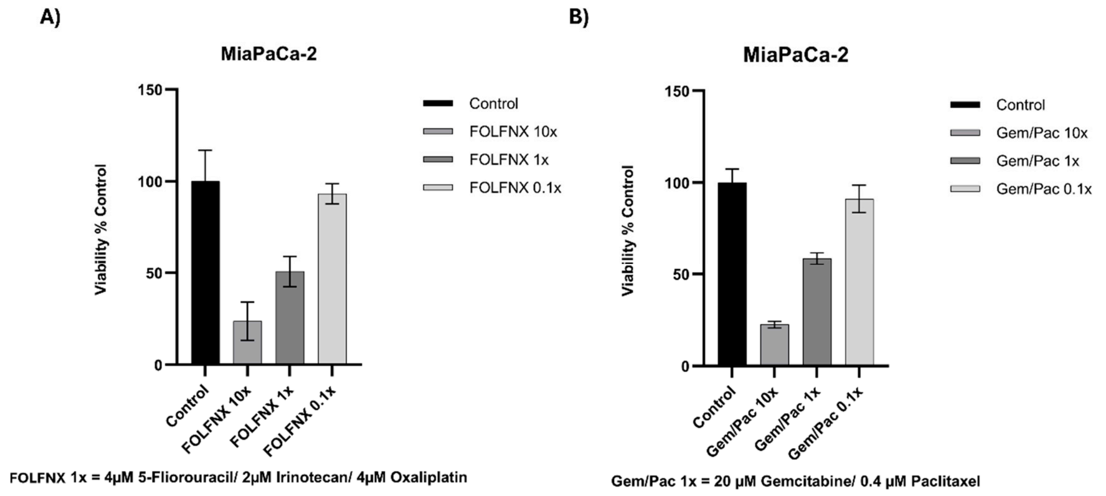

Supplementary Figure S1. Dose–response analysis for chemotherapy concentration selection in MIA PaCa-2 cells.

MIA PaCa-2 cells were treated for 24 hours with three relative concentrations (0.1x, 1x, and 10x of defined reference concentrations) of (A) FOLFNX (5-fluorouracil + irinotecan + oxaliplatin) or (B) GEMPAC (gemcitabine + paclitaxel). Reference (1x) concentrations corresponded to 4 μM 5-fluorouracil + 2 μM irinotecan + 4 μM oxaliplatin for FOLFNX and 20 μM gemcitabine + 0.4 μM paclitaxel for GEMPAC. Cell viability was assessed using the MTS assay (CellTiter 96® AQueous One Solution Cell Proliferation Assay, Promega). Both regimens demonstrated graded, concentration-dependent reductions in viability. The 1x concentration produced an intermediate (~40–60%) reduction in viability and was selected for downstream proteomic analysis.
